# Integration of Small- and Wide-Field Visual Features in Target-Selective Descending Neurons of both Predatory and Nonpredatory Dipterans

**DOI:** 10.1523/JNEUROSCI.1695-18.2018

**Published:** 2018-12-12

**Authors:** Sarah Nicholas, Jack Supple, Richard Leibbrandt, Paloma T. Gonzalez-Bellido, Karin Nordström

**Affiliations:** ^1^Centre for Neuroscience, Flinders University, 5001 Adelaide, South Australia, Australia,; ^2^Department of Ecology, Evolution, and Behavior, University of Minnesota, St. Paul, Minnesota 55108, and; ^3^Department of Physiology, Development and Neuroscience, University of Cambridge, Cambridge CB2 3EG, United Kingdom

**Keywords:** dipteran TSDN, motion vision, optic flow, target detection, target tracking

## Abstract

For many animals, target motion carries high ecological significance as this may be generated by a predator, prey, or potential mate. Indeed, animals whose survival depends on early target detection are often equipped with a sharply tuned visual system, yielding robust performance in challenging conditions. For example, many fast-flying insects use visual cues for identifying targets, such as prey (e.g., predatory dragonflies and robberflies) or conspecifics (e.g., nonpredatory hoverflies), and can often do so against self-generated background optic flow. Supporting these behaviors, the optic lobes of insects that pursue targets harbor neurons that respond robustly to the motion of small moving objects, even when displayed against syn-directional background clutter. However, in diptera, the encoding of target information by the descending neurons, which are more directly involved in generating the behavioral output, has received less attention. We characterized target-selective neurons by recording in the ventral nerve cord of male and female predatory *Holcocephala fusca* robberflies and of male nonpredatory *Eristalis tenax* hoverflies. We show that both species have dipteran target-selective descending neurons that only respond to target motion if the background is stationary or moving slowly, moves in the opposite direction, or has un-naturalistic spatial characteristics. The response to the target is suppressed when background and target move at similar velocities, which is strikingly different to the response of target neurons in the optic lobes. As the neurons we recorded from are premotor, our findings affect our interpretation of the neurophysiology underlying target-tracking behaviors.

**SIGNIFICANCE STATEMENT** Many animals use sensory cues to detect moving targets that may represent predators, prey, or conspecifics. For example, birds of prey show superb sensitivity to the motion of small prey, and intercept these at high speeds. In a similar manner, predatory insects visually track moving prey, often against cluttered backgrounds. Accompanying this behavior, the brains of insects that pursue targets contain neurons that respond exclusively to target motion. We here show that dipteran insects also have target-selective descending neurons in the part of their nervous system that corresponds to the vertebrate spinal cord. Surprisingly, and in contrast to the neurons in the brain, these premotor neurons are inhibited by background patterns moving in the same direction as the target.

## Introduction

Target detection and tracking serve important biological functions for animals to efficiently avoid predators, find prey, or identify conspecifics. Target detection can be performed by different senses. Bats find prey with echolocation (Falk et al., 2014), squid embryos avoid predators with their lateral line system (York et al., 2016), and insects visually identify conspecifics (Land and Collett, 1974). Often, targets need to be visualized against self-generated optic flow, which is a difficult computational task (Yang et al., 2012; Held et al., 2016), especially in conditions where both local luminance and relative contrast may change rapidly (Mohamed et al., 2014; Ma et al., 2015). Nonetheless, many insects appear to have solved this efficiently, as evidenced by their high-speed pursuits of targets, which is particularly impressive considering that insects carry low-spatial resolution compound eyes and small brains (Land, 1997).

Notably, many insects that pursue targets display local adaptations. For example, the eyes of target-pursuing insects often have faster photoreceptors (Weckström and Laughlin, 1995; Burton and Laughlin, 2003; Gonzalez-Bellido et al., 2011) and a region with increased spatial resolution, a fovea, in which they try to place the image of the target during aerial pursuits (Collett, 1980; Olberg et al., 2007; Wardill et al., 2017). The optic lobes harbor target-sensitive neurons with receptive fields that often collocate with the optical fovea (Strausfeld, 1980; Barnett et al., 2007), such as small target motion detector (STMD) neurons (O'Carroll, 1993; Nordström et al., 2006). STMDs could relay signals to target-selective descending neurons (TSDNs; Olberg, 1981, 1986; Gronenberg and Strausfeld, 1990; Namiki et al., 2018) whose receptive fields also colocate with the optical fovea (Gonzalez-Bellido et al., 2013).

In the lobula, some hoverfly and dragonfly STMDs show remarkably robust responses to targets moving in visual clutter (Nordström and O'Carroll, 2009). In fact, the STMD response to target motion is unaffected by the addition of background motion with the same velocity (i.e., syn-directional motion, without relative velocity differences; Nordström et al., 2006; Wiederman and O'Carroll, 2011). This can be modeled (Wiederman et al., 2008) using the associated unique spatiotemporal profile, where the dark contrast edge of the target (OFF) is immediately followed by a bright contrast change (ON). Models incorporating temporal correlation of half-wave rectified ON and OFF signals from the same point in space, together with fast adaptation and strong spatial inhibition, lead to robust responses to target motion in visual clutter (Wiederman et al., 2008). To date, it is largely unclear how TSDNs respond to targets moving in clutter (except for one data trace, see Olberg, 1986). This is important as TSDNs are closer to the motor output (Gonzalez-Bellido et al., 2013) and may thus be more pertinent for interpreting the behavioral relevance of neurophysiological recordings. In addition to the TSDNs, there are many other descending neurons (Namiki et al., 2018), such as those that respond to background motion (Wertz et al., 2009a, b). These project to areas of the thoracic ganglia with motor neurons of the neck, wings, and halteres (Suver et al., 2016).

As the ventral nerve cord forms an information bottleneck between the central processing of visually driven information and peripheral transformation into motor output (Namiki et al., 2018), we here quantified the response to moving targets and visual clutter. For this purpose, we characterized dipteran TSDNs (dTSDNs) in the predatory robberfly *Holcocephala fusca* and the nonpredatory hoverfly *Eristalis tenax*, the latter of which pursue conspecifics. As the two animals pursue targets for different reasons, and are evolutionarily distant, any similarities could potentially inform us about general mechanisms underlying dipteran target detection. In *Eristalis*, we also identified neurons sensitive to visual clutter. We found that *Holcocephala* and *Eristalis* dTSDNs did not respond to targets moving across background clutter, unless the background moved in the opposite direction, moved slowly, or was highly un-naturalistic, suggesting that dTSDNs receive inhibitory input from presynaptic neurons tuned to wide-field optic flow.

## Materials and Methods

### 

#### 

##### Animals and electrophysiology.

Thirty-eight male *E. tenax* hoverflies were reared from eggs laid by wild-caught hoverflies and housed as described previously (Nicholas et al., 2018). Sixteen female and 3 male adult *H. fusca* robberflies were wild caught and recorded from on the day of capture. Before recording, the animal was immobilized dorsal side down and a small hole was cut at the anteroventral thoracic surface to expose the ventral nerve cord.

For *Eristalis* extracellular recordings, a sharp polyimide-insulated tungsten electrode (0.1 MΩ; MicroProbes) was inserted into the nerve cord, with mechanical support given to the cord by a small wire hook. The animal was grounded via a silver wire inserted into the ventral cavity, which also served as the recording reference. To prevent the drying up of the exposed ventral cavity, a small amount of a petroleum jelly and mineral oil mix (1:1 ratio) was applied. Extracellular signals were amplified at 1000× gain and filtered through a 10–3000 Hz bandwidth filter on a DAM50 differential amplifier (World Precision Instruments), filtered through a HumBug (Quest Scientific), digitized via a PowerLab 4/30 (ADInstruments), and acquired at 10 kHz with LabChart 7 Pro Software (ADInstruments).

For *Holcocephala* extracellular recordings, a sharp glass-insulated tungsten electrode (2–4 MΩ; Microelectrodes) was inserted into the nerve cord, with mechanical support given to the cord by a small hook fashioned from a hypodermic needle. The animal was grounded by a saline-filled microelectrode inserted into the ventral cavity, which also served as the recording reference. Fly saline was prepared as follows (Gengs et al., 2002): 138 mm NaCl, 2 mm KCl, 1.8 mm CaCl_2_, 4 mm MgCl_2_, and 5 mm TES (triethylsilane), pH 7.15. Hydration of the ventral cavity was maintained by continual capillary action from an additional saline-filled microelectrode. Extracellular signals were amplified at 500× gain and filtered through a 300–3000 Hz analog bandpass filter on an NPI-BA Amplifier (NPI Electronic), filtered through a HumBug (Digitimer), digitized on a Micro1401 data acquisition unit (Cambridge Electronic Design), and acquired at 25 kHz with Spike2 Software (Cambridge Electronic Design).

##### Visual stimuli.

For *Eristalis* experiments, visual stimuli were displayed on an LCD screen (Asus) with a spatial resolution of 2560 × 1440 pixels running at 165 Hz, using the Psychophysics toolbox in Matlab (MathWorks 2017). *Eristalis* males were placed at a distance of 7 cm, giving a projected screen size of 154° × 137°. For *Holcocephala* experiments, visual stimuli were projected onto a 17.3 × 9.6 cm white screen using a DepthQ 360 Projector (Cambridge Research Systems) with a spatial resolution of 1280 × 720 pixels running at 360 Hz, using StimulateOpenGL Software (version 20160216, Janelia Research Campus, https://github.com/cculianu/StimulateOpenGL_II). *Holcocephala* animals were placed at a distance of 7 cm, giving a projected screen size of 102° × 70°.

**Figure 1.**
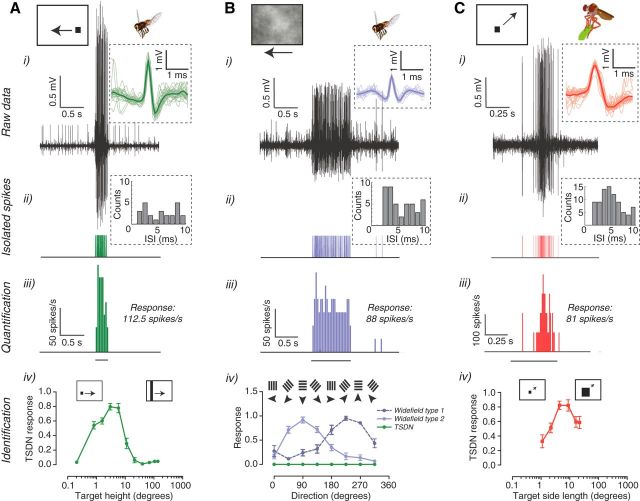
*Eristalis* and *Holcocephala* have dTSDNs. ***A***, ***i***, An example of a dTSDN response recorded in the *Eristalis* ventral nerve cord during stimulation with a small target drifted horizontally across its receptive field. The inset diagram shows the mean (thick line) and individual (thin lines) waveforms of 30 action potentials isolated from this example. ***ii***, The resulting spike train as a function of time. The inset graph shows the interspike intervals (ISIs). ***iii***, Histogram of spike rate within 40 ms bins and mean response during target motion across the dTSDN receptive field (bar under data). ***iv***, The response to different target heights, where the target width was fixed at 3°. When the bar subtended >10° of the visual field, the dTSDN response was strongly suppressed. The data are normalized to the maximum response of each neuron. *N* = 27. ***B***, ***i***, Example of wide-field neuron response recorded from the ventral nerve cord in *Eristalis* during stimulation with a background pattern drifting horizontally. The inset diagram shows the mean (thick) and individual (thin lines) waveforms of 30 action potentials. ***ii***, The resulting spike train as a function of time and the ISI (inset). ***iii***, The spike rate in 40 ms bins and the mean response during the peristimulus duration (bar under data). ***iv***, The response to high-contrast sinusoidal gratings moving in eight different directions (wavelength at 7°, 5 Hz) of two different types of wide field-sensitive neurons, here referred to as type 1 (dark purple, *N* = 8) and type 2 (light purple, *N* = 14), and of dTSDNs (green, *N* = 27). ***C***, ***i***, An example response of a *Holcocephala* TSDN and the waveform (inset). ***ii***, The resulting spike train and ISI. ***iii***, The spike rate within 10 ms bins and the mean response for the stimulus duration (bar under data). ***iv***, The response across neurons to square targets of varying size (side length indicated on *x*-axis). The target appeared at a random position on the screen, remained stationary for 150 ms, and then moved in a random direction for 100 ms. *N* = 12. The data are normalized to the maximum response of each neuron. In all panels, the data are displayed as the mean ± SEM.

For all dTSDN experiments, we first mapped the receptive field (Nordström et al., 2006; Gonzalez-Bellido et al., 2013) and confirmed that the neuron responded selectively to small targets and not to wide-field optic flow (Fig. 1) or to looming stimuli. For all target–background experiments, the targets moved either horizontally (*Eristalis*) or vertically (*Holcocephala*) across the center of the receptive field of each neuron. Only data from neurons showing both a robust and consistent response to the target moving over a gray background throughout the recording were included in this study. For size-tuning experiments in *Eristalis*, we moved a black target with a fixed width (3°) horizontally across a white background at an average velocity of 180°/s (since we used flat screens, the projected angular velocity varied between their central and peripheral parts). We varied the vertical extent of the target from the smallest we could display on the screen (0.2°) to bars that covered its entire height (137°). For size-tuning experiments in *Holcocephala*, a small black square target appeared in a random location anywhere on the screen, remained stationary for 150 ms, and then moved in a random direction for 100 ms, in a total of 2400 random trajectories (Gonzalez-Bellido et al., 2013).

For target–background experiments in both *Eristalis* and *Holcocephala*, we used an artificially generated naturalistic background pattern with a slope constant (α) of the amplitude spectrum and rms contrast close to those of natural scenes (Dyakova and Nordström, 2017). To generate this background pattern, we used the fact that the spatial statistics of an image can be quantified by constructing a fast Fourier transform and plotting the rotationally averaged amplitude as a function of spatial frequency (Tolhurst et al., 1992). Displayed in a log-log graph, the amplitude is then inversely proportional to the spatial frequency raised to the power α (Dyakova and Nordström, 2017). In natural scenes, the α is close to 1.1 (Tolhurst et al., 1992; Dyakova and Nordström, 2017), which we used here. We set the mean luminance of the background to the mean of the screen and always covered the entire screen. In *Eristalis*, the target (3° × 3°) moved horizontally across the screen at an average velocity of 180°/s. In *Holcocephala*, the target (2° × 2°) moved vertically across the screen at an average velocity of 160°/s. Unless otherwise indicated, the target and the background moved at the same velocity.

In *Eristalis*, we defined optic flow-sensitive neurons based on their receptive field properties and response to a high-contrast sinusoidal grating moving in eight different directions (wavelength of 7° drifting at 5 Hz; Fig. 1*Biv*), using blowfly data as a comparison (Wertz et al., 2009a,b). We displayed the same artificially generated naturalistic background pattern as in the target–background experiments described above, but without the target. The pattern moved horizontally across the screen, unless otherwise indicated.

*Holcocephala* recording time was limited due to the animals having to be used on the day of capture, and only being available for a maximum of 8 weeks of the year. Therefore, we were able to explore more stimulus parameters in *Eristalis*. We recorded from 27 dTSDNs and 29 neurons sensitive to wide-field motion in 38 male *Eristalis* and from 38 dTSDNs in 3 male and 16 female *Holcocephala*. In all experiments, in both species, the stimulus trial order was randomized.

##### Experimental design and statistical analysis.

Spike sorting of *Eristalis* extracellular data (Fig. 1*Ai*,*Bi*) was performed using LabChart 7 Pro with the Spike Histogram Add-On (ADInstruments), which uses the action potential amplitude and width to identify responses from individual neurons (Fig. 1*Ai*,*Bi*,insets). In addition, we quantified the interspike intervals (Fig. 1*Aii*,*Bii*) from the resulting spike trains. All further data analysis was performed in Matlab. *Holcocephala* extracellular data (Fig. 1*Ci*) were sorted in Spike2 (Cambridge Electronic Design), which uses principal component analysis on the waveform shape (Fig. 1*Ci*, inset) followed by manual clustering.

For all experiments in *Eristalis* TSDNs, we quantified the mean spike frequency for the time that the target traversed the receptive field of each neuron (Fig. 1*Aiii*, bar under data). For optic flow-sensitive neurons, we quantified the mean spike frequency for the entire stimulus duration (Fig. 1*Biii*, bar under data). The dTSDNs were not spontaneously active (Fig. 1*A*), but the optic flow-sensitive neurons sometimes had a spontaneous rate, which is indicated in each graph. All experiments were repeated 6–18 times in each animal, where we varied the precise target location slightly between trials to avoid habituation. The data from repetitions within a neuron were averaged, with the graphs in the article showing variation across neurons.

*Holcocephala* and *Eristalis* responses to differently sized targets were normalized to the maximum response of each neuron. To separate target-induced responses from background-induced responses in *Holcocephala*, the response to the same time window when only a background pattern was shown was subtracted from the response to target only motion (Fig. 1*Ciii*, bar under data). For *Holcocephala* target–background experiments, each condition consisted of three repetitions of the stimuli. Due to the fast habituation of the neurons in *Holcocephala*, only the response from the first presentation of the stimuli from each animal was used for further analysis.

Statistical analysis was performed in GraphPad Prism (version 7.0d, GraphPad Software), after ensuring that the data were normally distributed, with details of the test and significance given in each figure legend. *p* values <0.05 were used to refute the null hypothesis.

## Results

### Descending neuron identification

We performed extracellular recordings in the ventral nerve cord of *H. fusca* robberflies and *E. tenax* hoverflies (Fig. 1, top row). We identified individual neurons based on the waveform (including amplitude and width of each action potential; Fig. 1, insets, top row). We defined descending visual neurons as dTSDNs by their peak response to small objects subtending a few degrees of the visual field, with no response to elongated bars (*Eristalis* data; Fig. 1*Ai*,*iv*), to larger objects (*Holcocephala* data, Fig. 1*Ci*,*iv*), and to looming or wide-field stimuli (*Eristalis* data; Fig. 1*Biv*). The selective response to the motion of small targets in these dTSDNs (Fig. 1) is similar to the response properties of previously described STMDs found in the lobula of *Eristalis* hoverflies (Nordström et al., 2006). These results (Fig. 1*A*,*C*) are in accordance with the previously proposed notion that TSDNs may be downstream of the lobula STMD neurons (Barnett et al., 2007; Nordström and O'Carroll, 2009). Whether these neurons are directly or indirectly connected remains to be clarified.

In *Eristalis*, we identified a second group of wide field-sensitive descending visual neurons, which respond to sinusoidal gratings in a direction-selective manner (Fig. 1*B*). We predominantly recorded from two types of wide field-sensitive neurons, which responded preferentially to motion up and to the right across the visual field of the animal (Fig. 1*Biv*, 225°, light purple) and to motion down across the visual field (Fig. 1*Biv*, 90°, dark purple), respectively. The direction tuning (Fig. 1*Biv*) follows the typical sinusoidal shape seen in similar descending neurons previously described in blowflies and *Drosophila*, which receive direct input from optic flow-tuned lobula plate tangential cells (LPTCs; Wertz et al., 2009b; Suver et al., 2016).

### Dipteran TSDNs do not respond to targets moving in the same direction as background clutter

**Figure 2.**
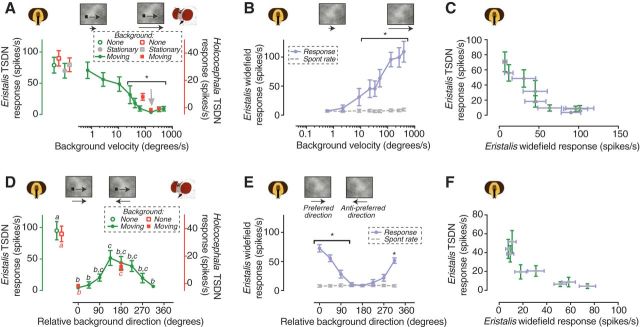
Syn-directional background pattern motion strongly suppresses the dTSDN response to target motion. ***A***, The dTSDN response to a small target drifted across its receptive field. The target was presented against a naturalistic background pattern moving in the same direction as the target. The green data show the response of *Eristalis* TSDNs (*N* = 8), and the red data the response of *Holcocephala* TSDNs (*N* = 38). *p < 0.05, one-way ANOVA. ***B***, The response of *Eristalis* wide field-sensitive neurons to the naturalistic background pattern moving at different velocities (*N* = 8). Significant differences between response and spontaneous rate are indicated (two-way ANOVA followed by Sidak's multiple-comparisons test). ***C***, The *Eristalis* TSDN response as a function of the response of the wide field-sensitive neurons when using background patterns moving at different velocities. ***D***, The dTSDN response to a small target drifted across its receptive field. The target was presented against a naturalistic background pattern moving at the same speed as the target, but in different directions. The green data show the response of *Eristalis* TSDNs (*N* = 5), and the red data the response of *Holcocephala* TSDNs (*N* = 38). Different letters above (*Eristalis*) or below (*Holcocephala*) the data points indicate that they are significantly different from each other (one-way ANOVA followed by Tukey's multiple-comparisons test, tested separately for each species). ***E***, The response of *Eristalis* wide field-sensitive neurons to the naturalistic background pattern moving in different directions (*N* = 8). Significant differences between response and spontaneous rate are indicated (two-way ANOVA followed by Sidak's multiple-comparisons test). ***F***, The response of the *Eristalis* TSDNs as a function of the response of the wide field-sensitive neurons when the background pattern moved in different directions. In all panels, the data are displayed as the mean ± SEM.

In the *Eristalis* lobula, some STMDs respond robustly to targets moving in visual clutter, even when there are no velocity differences between target and background (Nordström et al., 2006). To investigate whether this property is also present at the dTSDN level, we presented *Eristalis* and *Holcocephala* with a small, high-contrast target (3° × 3° and 2° × 2°, respectively) moving across a background pattern. The background was artificially generated to have naturalistic spatial statistics and rms contrast (Dyakova and Nordström, 2017). The target was presented moving horizontally (*Eristalis*) or vertically (*Holcocephala*) across the screen, with the background pattern moving in the same direction. As controls, we recorded the response to the target moving over a mean luminance background (Fig. 2*A*, open symbols) or over the cluttered background presented stationarily (Fig. 2*A*, gray symbols, gray symbols). The responses to these two controls were not significantly different from each other (Fig. 2*A*).

We found a consistent trend in both species, as follows: the presence of background movement substantially reduced the dTSDN responses (Fig. 2*A*). This effect became significant when the background moved at velocities >10°/s (Fig. 2*A*, green data). When the target and the background moved at the same velocity (Fig. 2*A*, gray arrow), the response to the motion of the target had completely disappeared in both species (Fig. 2*A*, *Eristalis* = green, *Holcocephala* = red). The dTSDN responses were also absent when the background moved two or three times faster than the target (Fig. 2*A*, data points to the right of gray arrow) or at half the velocity in *Eristalis* (Fig. 2*A*, green data point to the left of the gray arrow). It thus seems as if the dTSDNs are unresponsive to targets presented against syn-directional background motion, with or without relative velocity differences.

We next recorded from optic flow-sensitive neurons in the *Eristalis* descending nerve cord. We found that the response of the wide-field neurons increased with the velocity of the background pattern (Fig. 2*B*). We also found that as the response of the *Eristalis* wide-field neurons to background velocity increased, the response of the dTSDNs to targets moving over backgrounds with different velocities decreased (Fig. 2*C*, Table 1, different curve fits to the data).

**Table 1.** Fit parameters for the data shown in Figures 2, *C* and *F*, 3, *C* and *F*, and 4*C*

Background variable	*f*(*x*) = *k* + *c* * *x*		*f*(*x*) = *k* + *c/x*		*f*(*x*) = *k* + *c/x^2^*		*f*(*x*) = *k* * e^-c^*^x^	
	Coefficient of determination (*R*^2^)	SD of the residuals (Sy.x)	*R*^2^	Sy.x	*R*^2^	Sy.x	*R*^2^	Sy.x
Velocity (Fig. 2*C*)	0.8411	10.46	0.7888	12.06	0.6426	15.69	0.9347	7.244
Direction (Fig. 2*F*)	0.7879	9.062	0.8469	7.697	0.7351	10.13	0.8859	7.281
Cover (Fig. 3*C*)	0.7242	8.753	0.9845	2.077	0.9893	1.727	0.9881	2.033
Height (Fig. 3*F*)	0.7753	10.44	0.9247	6.044	0.9358	5.581	0.9306	6.701
Contrast/alpha (Fig. 4*C*)	0.9132	8.899	0.8197	12.2	0.6151	18.73	0.9762	5.027

For each data set we show two fit parameters, which can be used to determine how well the data fit a particular function. The coefficient of determination (*R*^2^) quantifies the goodness of the fit on a scale from 0 to 1 (perfect), and the standard deviation of the residuals (Sy.x) is similar to the root mean square error (RMSE) but the number of parameters fit by the regression are taken into account. In all conditions, we used a least squares, unconstrained fit, with a maximum of 1000 iterations.

### Dipteran TSDNs respond stronger to target motion when the background moves in the opposite direction

Next, we tested whether the direction of target and background motion were important in their interactions. We presented the target and background moving at the same speed (Fig. 2*A*, gray arrow), but tested a variety of background directions (in steps of 45°; Fig. 2*D*, closed symbols). For comparison, we recorded the response to targets moving over a uniform mean luminance background (Fig. 2*D*, open symbols). We found that the response to target motion depended on the direction of background motion. As shown above, the response was completely suppressed when the target and the background moved in the same direction (i.e., 0° relative direction difference; Fig. 2*D*). The dTSDN response to the motion of the target increased when the background moved in the opposite direction to the target (i.e., 180° relative direction difference; Fig. 2*D*). However, even when the background moved in the opposite direction to the target, the response was significantly lowered to 46% in *Eristalis*, and 24% in *Holcocephala* compared with the control condition (no background; Fig. 2*D*), showing remarkable consistency across the two species. Importantly, the *Eristalis* response was strongly suppressed even when the background moved at a relative direction of 45° (Fig. 2*D*, green data).

In *Eristalis*, we next recorded the response of descending wide field-sensitive neurons to the background pattern moving in different directions (Fig. 2*E*). In each recording, we defined 0 as the preferred direction of the neuron (Fig. 1*Biv*, for the underlying direction preferences of the two most commonly encountered wide field-sensitive neurons). Again, we found that the response of the *Eristalis* TSDNs to targets moving over backgrounds in different directions decreased as the response of the wide-field neurons to different directions of background motion increased (Fig. 2*F*, Table 1).

### Local mechanisms do not explain dTSDN response suppression from background motion

In some STMDs in the *Eristalis* brain, a moving target is detected against background motion even in the absence of relative movement (Nordström et al., 2006), but this ability seems to be gone in the dTSDNs (Fig. 2). Thus, our dTSDN and wide-field descending neuron results (Fig. 2) led us to postulate that the response to target motion is actively suppressed by the wide-field system. The suppression appears to be weaker at low velocities (Fig. 2*A*,*C*) and when the background moves in a different direction to the target (Fig. 2*D*,*F*).

**Figure 3.**
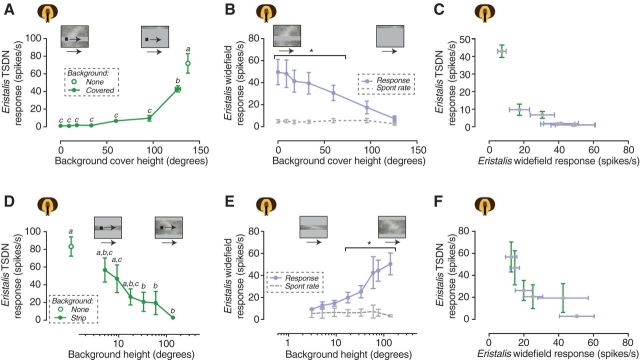
A small amount of background pattern motion suppresses the dTSDN response to target motion. ***A***, The *Eristalis* TSDN response to a small target moving across a background pattern, both moving at the same velocity. The background pattern was covered by a stationary mean luminance strip of different heights, centered on the trajectory of the target. Different letters indicate that the data points are significantly different from each other (one-way ANOVA followed by Tukey's multiple-comparisons test, *N* = 7). ***B***, The response of *Eristalis* wide field-sensitive neurons to the background pattern, covered by a stationary mean luminance strip of different heights, centered on the receptive field of each neuron. *N* = 7. Significant differences between response and spontaneous rate are indicated (two-way ANOVA followed by Sidak's multiple-comparisons test). ***C***, The response of the *Eristalis* TSDNs as a function of the response of the wide field-sensitive neurons when the background pattern was covered by a gray strip. ***D***, The *Eristalis* TSDN response to a small target moving across a background pattern, at the same velocity. The vertical extent of the background pattern was varied, and centered on the trajectory of the target. Different letters indicate that the data points are significantly different from each other (one-way ANOVA followed by Tukey's multiple-comparison's test, *N* = 7). ***E***, The response of *Eristalis* wide field-sensitive neurons to different background pattern heights. *N* = 11. Significant differences between response and spontaneous rate are indicated (two-way ANOVA followed by Sidak's multiple-comparisons test). ***F***, The response of the *Eristalis* TSDNs as a function of the response of the wide field-sensitive neurons when the vertical extent of the background pattern was varied. In all panels, the data are displayed as the mean ± SEM.

One possible alternative explanation is that the response suppression is caused by a reduced local relative contrast associated with the target moving over the naturalistic background, compared with when it moves over a uniform mean luminance background (Fig. 2*A*,*D*, open symbols). To investigate this possibility, we placed a gray mean luminance patch over the background, centered on the trajectory of the target. The patch ensured that the local contrast surrounding the trajectory of the target was equal to the control condition (Fig. 2*A*,*D*, open symbols). For this experiment, the target and the background moved at the same velocity. We found that a small cover did not increase the dTSDN response (the smallest cover was three times higher than the target; Fig. 3*A*, closed symbols). It was not until we covered the majority of the background texture (127° height, 77% of the total area) that the dTSDN response to target motion increased significantly, but even then it was significantly lower than under control conditions (Fig. 3*A*). This was surprising because in all these cases the local contrast was the same as in the control condition (Fig. 3*A*, open symbol).

We next recorded the response of wide field-sensitive neurons to the background pattern when it was covered by a similar gray mean luminance “strip” of varying heights. We found that the response of the wide-field neurons decreased linearly with the height of the cover (Fig. 3*B*). In addition, we found that the response of the *Eristalis* dTSDNs to different cover heights decreased as the response of the wide-field neurons increased when the background texture was covered (Fig. 3*C*, Table 1).

To test whether the extent of the background pattern matters, we followed up by inverting the plain and background areas: the strip surrounding the target now contained the original background pattern, and the screen outside of the patch had uniform luminance (i.e., no pattern). We varied the size, and thus the percentage of screen covered, with a moving, patterned background. We found that the dTSDN response to target motion decreased as the height of the patch with the moving background pattern increased. However, for the drop in dTSDN responses to reach significance, the patterned patch had to cover 70% of the screen height (while the projected target covered only 1% of the height; Fig. 3*D*). By recording from wide field-sensitive neurons, we found that the wide-field response increased with the height of the background pattern (Fig. 3*E*), and that the response of the *Eristalis* TSDNs decreased as the response of the wide-field neurons increased (Fig. 3*F*, Table 1).

Together, these experiments (Fig. 3) suggest that it is not local contrast differences between the target and the background that result in the dTSDNs being silent when the target and background move in the same direction. Rather, it seems as if optic flow information inhibits the dTSDNs.

### Dipteran TSDN response suppression is strongest when the background is most naturalistic

**Figure 4.**
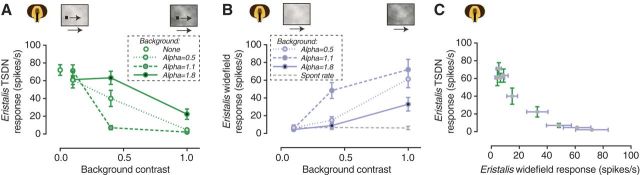
Target motion is least visible when the background pattern is most naturalistic. ***A***, The *Eristalis* TSDN response to a small target moving across a background pattern, at the same velocity. We varied the contrast of the background pattern (*x*-axis) and the slope constant of its amplitude spectrum (α, as color coded). A two-way ANOVA showed a significant effect of α (*p* = 0.0046) and contrast (*p* < 0.0001), and a significant interaction between contrast and α (*p* = 0.0002). *N* = 5. ***B***, The response of *Eristalis* wide field-sensitive neurons to background patterns with varied contrast (*x*-axis) and a slope constant of its amplitude spectrum (α, as color coded). A two-way ANOVA showed a significant effect of α (*p* < 0.0001) and contrast (*p* < 0.0001), and a significant interaction between contrast and α (*p* < 0.0001). *N* = 7. ***C***, The response of the *Eristalis* TSDNs as a function of the response of the wide field-sensitive neurons when we varied background pattern contrast and slope constant. In all panels, the data are displayed as the mean ± SEM.

The background pattern used in our experiments was artificially generated to have natural image statistics with respect to its contrast and amplitude spectrum (Dyakova and Nordström, 2017). Natural images have amplitude spectra slope constants of nearly 1 (Tolhurst et al., 1992), to which both peripheral and central sensory neurons are tuned (van Hateren, 1992; Song and Juusola, 2014; Dyakova et al., 2015). As a final experiment, we varied the following two background properties: the contrast of the pattern and the slope constant of its amplitude spectrum. The target and the background moved at the same velocity (i.e., there was no relative motion between the two). We found that the dTSDN responses to target motion were higher when the contrast of the background was lower (Fig. 4*A*). In addition, when the background had medium contrast (0.4), the most naturalistic pattern (α, 1.1), resulted in a near absence of dTSDN responses (Fig. 4*A*, dashed line, gray symbol). This was surprising because with the same contrast level, the α of 1.8 gave responses similar to those for controls, and an α of 0.5 reached at least half of the control responses. This finding is of importance because it suggests that the suppression of dTSDN responses, shown in this study, is strongest when the target is presented against more naturalistic backgrounds.

We recorded the response of *Eristalis* wide-field neurons to the same background patterns and found that the response increased with contrast (Fig. 4*B*). Furthermore, at medium background contrast (0.4) the strongest response was generated by the most naturalistic background pattern (Fig. 4*B*, dashed line, gray symbol, α of 1.1). Again, the dTSDN response decreased as the response of the optic flow neurons increased when we varied the contrast and amplitude spectrum of the background pattern (Fig. 4*C*, Table 1). Thus, our findings are consistent with the hypothesis that wide field-sensitive neurons are involved in suppressing the dTSDN responses to target motion.

## Discussion

We have shown that *Eristalis* and *Holcocephala* have dTSDNs (Fig. 1) with similar size tuning as STMDs in the dragonfly and hoverfly lobula (O'Carroll, 1993; Nordström et al., 2006) and dragonfly TSDNs (Olberg, 1986; Gonzalez-Bellido et al., 2013). dTSDNs thus show physiological homology to dragonfly TSDNs, but whether they are also morphological homologs remains to be studied. dTSDNs are unresponsive to targets moving in visual clutter (patterned background), unless the background is moving slowly (Fig. 2*A*) or in the opposite direction (Fig. 2*D*), or has un-naturalistic spatial characteristics (Fig. 4*A*). In *Eristalis*, we also recorded from neurons that respond to wide-field optic flow, with response properties similar to those of descending neurons described in blowflies and *Drosophila* (Wertz et al., 2009b; Suver et al., 2016). In all tested conditions, we found that background stimuli that increased the activity in the wide-field descending neurons led to the dTSDN responses decreasing (Figs. 2*C*,*F*, 3*C*,*F*, 4*C*, Table 1). Our findings are important as dTSDNs act as a bottleneck between sensory processing and behavioral motor output. Our results demonstrate that dTSDN response suppression induced by background motion is (1) not explained by local effects (Fig. 3) and (2) is likely caused by presynaptic neurons tuned to wide-field optic flow.

### Target-selective neurons

We defined *Eristalis* and *Holcocephala* neurons as dTSDNs based on their sharp size selectivity, with no response to full-screen bars, large objects, or sinusoidal gratings that drive optic flow-sensitive neurons strongly (Fig. 1). Dragonfly TSDNs descend from the brain (Olberg, 1986) and project to the subesophageal ganglion and all three thoracic ganglia (Gonzalez-Bellido et al., 2013), where they likely connect with motor neurons of the forewings and the hindwings. In dipterans, descending visual neurons project to the three thoracic ganglia, where they may control neck, leg, and/or wing motor neurons (Namiki et al., 2018). The dTSDNs described here are thus likely to provide input to the motor neurons involved in target–pursuit behaviors, by, for example, aligning the head (and thus the fovea) to the image of the target, and/or by rapidly changing flight course as is necessary during high-speed target pursuits (Collett and Land, 1975, 1978; Wardill et al., 2017).

Some target neurons (STMDs) in the lobula respond robustly to the motion of small targets against a cluttered background, even without relative velocity differences (Nordström et al., 2006; Wiederman and O'Carroll, 2011). It was therefore surprising to find that the dTSDN responses (i.e., the responses of the presumed downstream targets of STMDs; Nordström and O'Carroll, 2009) were so strongly affected under similar stimulus conditions (Fig. 2*A*,*D*). Since the background inhibition was observed in both *Eristalis* and *Holcocephala* (Fig. 2*A*,*D*), whose responses were recorded at different times, by different teams, and with different instrumentation, this finding is clearly not a species- or experimental-specific oddity. Importantly, the target size, as well as the angular velocities of the target and the background were within the range of those experienced during target pursuits in *Eristalis* (Collett and Land, 1978) and *Holcocephala* (Wardill et al., 2017).

In hoverflies and other nonpredatory flies, target detection is primarily used for conspecific identification or territorial interactions (Wellington and Fitzpatrick, 1981), whereas predatory robberflies would use these neurons for the detection of suitable prey (Wardill et al., 2017). However, both species intercept their targets, with *Holcocephala* recently shown to use proportional navigation, a strategy that is likely shared by other dipterans (Fabian et al., 2018). Despite the marked ecological differences, the size tuning (Fig. 1*Aiv*, *Civ*) and responses to targets in clutter (Fig. 2*A*,*D*) were remarkably similar, suggesting that task-specific constraints resulted in the evolution of target-selective neurons with similar response properties across species.

### Background suppression

We found that in the presence of a moving background dTSDNs responded to the target only when the background velocity was low (Fig. 2*A*) or had un-naturalistic spatial characteristics (Fig. 4*A*); i.e., under conditions where the background clutter did not drive optic flow-sensitive neurons strongly; (Figs. 2*B*, 4*B*). The relationship between the responses of dTSDNs and the neurons tuned to wide-field optic flow (Figs. 2*C*,*F*, 3*C*,*F*, 4*C*, Table 1) suggests that activation of the wide-field pathway results in inhibition of the target-tracking pathway. At this stage, we are not able to pinpoint the type of inhibition as the relationship between the responses of wide-field neurons and dTSDNs could be described by either linear or nonlinear functions (Table 1). Since some STMDs in the lobula respond strongly under similar target–background conditions (Nordström et al., 2006; Wiederman and O'Carroll, 2011), it is possible that the inhibition takes place postsynaptic to these STMDs, but presynaptic to the dTSDNs. The observed inhibition could be implemented directly by the LPTCs since information from the lobula plate (wide-field motion) and the lobula (target motion) interacts extensively in the protocerebrum (Namiki et al., 2018). LPTCs have wide-reaching output synapses in the posterior slope (Suver et al., 2016), where they may synapse with dTSDNs. At least in dragonflies, TSDN input dendrites branch in this area (Olberg, 1986).

Suppression from the wide-field system was observed when the target moved over a moving background partially covered by a patch of uniform luminosity (Fig. 3*A*). Thus, it is unlikely that local contrast mechanisms related to target detection underpin our finding. As some STMDs respond under similar target–background conditions (Nordström et al., 2006), it is more likely caused by the suppression of responses to an already detected target.

Why go through all the trouble of extracting a clean target motion signal in the STMDs (Nordström et al., 2006) and then not transform it into a premotor command in the dTSDNs (Figs. 2, 3, 4)? One possibility is that during actual target pursuits, the target and the background only rarely move in the same direction. Indeed, during pursuits in the hoverfly *Syritta pipiens* the target is actively foveated, but when the target image leaves the fovea, the hoverfly performs saccadic tracking to reduce the target position error (Collett, 1980). During perfect foveation, there would be no remaining target motion, only background motion. One way to investigate this hypothesis would be to replay reconstructed target pursuits (Wardill et al., 2017). Furthermore, in highly textured environments *Holcocephala* and *Eristalis* use behavioral adaptations to eliminate the influence of background motion. For example, *Holcocephala* attempt to visualize the target against the clear sky (Wardill et al., 2017), whereas hoverflies often detect the target from a hovering stance, resulting in minimal background motion. In dragonflies, the prey detection distance is decreased by about one-third when prey is visualized against close background vegetation compared with distant background vegetation, respectively (Switzer and Eason, 2000), whereas blowfly target-tracking performance is unaffected by background motion (Trischler et al., 2010).

Another possibility is that our results are affected by our animals being immobilized, as visual responses are highly affected by the activity state of the fly (Maimon et al., 2010; Jung et al., 2011; Kim et al., 2015). Indeed, many descending neurons are multimodal (Tsubouchi et al., 2017), and even if TSDNs are predominantly visual (Olberg, 1986), they may be strongly affected by input from other modalities (Huston and Krapp, 2009; Kim et al., 2015; Fujiwara et al., 2017). Furthermore, during high-speed pursuit the fly suppresses stabilizing optomotor responses, which would otherwise counteract voluntary turns toward the target (Collett, 1980; Pal, 2015). Indeed, in flying *Drosophila* the prediction of the expected reafferent signal is quantitatively subtracted from some LPTCs in anticipation of voluntary turns (Kim et al., 2015). Assuming that this is also the case in our model system, LPTCs would not be able to inhibit the dTSDNs as observed in our data (Figs. 2, 3, 4). In blowflies, an artificial background rotation during conspecific pursuit does not alter the quality of chasing performance (Trischler et al., 2010), indicating that target tracking is unaffected by efference copy signals. Investigating these issues will require recording from dTSDNs under actual pursuit with telemetric recordings, as is already possible in larger insects (Fotowat et al., 2011; Harrison et al., 2011). This will be informative as *Drosophila* LPTCs and bee optic lobe neurons respond differently to the same visual stimulus experienced in open or closed loop (Paulk et al., 2014; Fujiwara et al., 2017).
